# Nutritional Status in Children with Un-Operated Congenital Heart Disease: An Egyptian Center Experience

**DOI:** 10.3389/fped.2015.00053

**Published:** 2015-06-15

**Authors:** Basheir A. Hassan, Ehab A. Albanna, Saed M. Morsy, Ahmed G. Siam, Mona M. Al Shafie, Hosam F. Elsaadany, Hanan S. Sherbiny, Mohamed Shehab, Oswin Grollmuss

**Affiliations:** ^1^Pediatrics Department, Faculty of Medicine, Zagazig University, Zagazig, Egypt; ^2^Pediatrics, Faculty of Medicine, INSERM 999, University of Paris-Sud, Orsay, France

**Keywords:** malnutrition, heart surgery, therapeutic options, congenital heart disease, Egyptian children

## Abstract

**Background:**

Malnutrition is a common cause of morbidity and mortality in children with congenital heart disease (CHD). This study aimed to identify prevalence and predictors of malnutrition in Egyptian children with symptomatic CHD.

**Methods:**

This case–control study included 100 children with symptomatic CHD (76 acyanotic and 24 cyanotic) and 100 healthy children matched for age and sex as a control group. Clinical Evaluation and Laboratory Assessment of Nutritional Status were documented. Anthropometric measurements were recorded and *Z* scores for weight for age (WAZ), weight for height (WHZ), and height for age (HAZ) have been calculated. Malnutrition was defined as weight, height, and weight/height *Z* score ≤–2.

**Results:**

The overall prevalence of malnutrition was 84.0% in patients with CHD and 20% in controls. Severe malnutrition was diagnosed in 71.4% of cases. All anthropometric measurements and levels of biochemical markers of nutritional state were significantly lower in the patients group compared to controls. In patients with acyanotic CHD, stunting was proportionately higher (57.89%) than in cyanotic CHD, while wasting was predominant (45.83%) in the latter. Malnutrition correlated significantly with low hemoglobin level, low arterial oxygen saturation, heart failure, pulmonary hypertension, and poor dietary history.

**Conclusion:**

Malnutrition is a very common problem in children with symptomatic CHD and predicted by the presence of low hemoglobin level, low arterial oxygen saturation, heart failure, poor dietary history, and pulmonary hypertension.

## Introduction

Congenital Heart Disease (CHD) are often associated with malnutrition and failure to thrive (1). Growth failure has been calculated with a prevalence of 64% in CHD patients in developed countries (2) being more severe in the developing regions, where malnutrition is common even in otherwise healthy children (3, 4).

Mechanisms for growth deficiency in CHD are multifactorial including associated chromosomal anomalies/genetic syndromes, inadequate nutrition due to feeding difficulties, and poor nutritional absorption from the digestive tract in chronic congestive heart failure (CHF). Also, increased caloric support is required to sustain the increased myocardial, respiratory, and neuro-humoral functions in CHD-related heart failure. Chronic CHF and chronic under-oxygenation in CHD impair cellular metabolism and cell growth, while repeated chest infections demand an increased metabolism (5).

Malnutrition in children with CHD has been associated with increased morbidity and mortality as indicated by frequent hospitalization, poor surgical outcomes, persistent impairment of somatic growth, and increased death (6, 7).

In Egypt, as in many developing countries, pediatric cardiac programs are not fully established and epidemiological data on CHD-related malnutrition are lacking. Therefore, this study aimed to assess the prevalence, patterns, and predictors of malnutrition in Egyptian children with symptomatic CHD.

## Patients and Methods

This study was conducted prospectively as a case–control study at the Pediatric Cardiology Unit, Zagazig University Children’s Hospital during the period from June 2012 to June 2013. It included 100 children aging from 2 months to 6 years with different symptomatic CHD. Patients with other congenital malformations than cardiac anomalies or other diseases (including genetic disorders) affecting the growth or the nutritional status of the children, were excluded.

One hundred healthy children matched in age and sex without any abnormal echocardiographic findings represented our control group.

Informed consent was obtained from the children’s parents or care-givers, and the study was approved by the ethical committee of faculty medicine of Zagazig University.

A full medical history as well as a complete cardiac examination (including Modified Ross’s Clinical score for heart failure) is documented for all children. Clinical signs of malnutrition, such as symmetrical edema, skin lesions, and dry, thin and depigmented hair, were assessed.

Anthropometric measurements included weight (kilogram), length (centimeter), cephalic, thoracic, and abdominal circumferences (centimeter) as well as triceps and subscapular skinfold thickness (millimeter). They were performed according to standard WHO procedures (8).

*Z* scores for weight for age (WAZ), weight for height (WHZ), and height for age (HAZ) were calculated using the Anthropometric calculator module of WHO Anthro software (version 3.2.2, January 2011) (based on The 2006 WHO child growth standards) (9).

After calculation of WAZ, WHZ, and (HAZ), the studied groups were classified into normal nutritional status and malnutrition (moderate or severe).

The WHO global database on child growth and malnutrition (under-nutrition) recommends a cut-off *Z* score of ≤-2 to classify low WAZ (underweight), low HAZ (stunting), and low WHZ (wasting) as moderate malnutrition, and a *Z* score of ≤-3 SD to define severe malnutrition (9).

Laboratory examinations included hemoglobin, serum protein, serum iron, and ferritin levels. Echocardiography was performed using the GE Vivid-7 multipurpose system with different probe sizes according to the recommendations of the American Society of Echocardiography (10).

### Statistical analysis

Data were analyzed using the Statistical Package for Social Sciences (SPSS) release 16. Data with a normal distribution are presented as mean and SD. The mean of two groups was compared by *t*-test. Non-parametric values were represented as median and range, and the medians of two groups were compared by Mann–Whitney *U* test. Qualitative data were represented by their frequency and relative percentage, and chi-square test was used for testing association of qualitative data. Correlations were performed using the Pearson bivariate correlation. A *p*-value of <0.05 was considered statistically significant.

## Results

A total of 100 children with symptomatic CHD were included in the study. Details of demographic characteristics, anthropometric measurements, and biochemical markers are given in Table 1. There were no significant differences between patients and controls regarding the demographic data except for birth weight, which was higher in the control group. Further, the poor nutritional history is significantly different in the patient cohort.

**Table 1:** **Demographic criteria, anthropometric measurements, and biochemical markers in patients and controls**.

	Cases (no. = 100)	Controls (no. = 100)	*p*-Value
**Demographic criteria**
Age (months)	12 (2–68)	14 (3–72)	>0.05^a^
Gender (males/females)	(44/56)	(47/53)	>0.05^b^
Gestational age (weeks)	39.5 ± 0.85	39.4 ± 0.88	>0.05^c^
Birth weight (kg)	2.7 ± 0.6	3.0 ± 0.5	<0.05^c^
Family size including parents	3.6 ± 0.7	3.8 ± 0.8	>0.05^c^
Poor nutritional history	68%	23%	<0.05^b^
**Anthropometric measurements**
Weight (kg), median (range)	7.5 (3–14)	10 (4.5–20)	<0.05^b^
Height or length (cm), mean ± SD	66.3 ± 4.3	74.7 ± 5.2	<0.05^c^
Head circumference (cm), mean ± SD	42.0 ± 4.1	44.3 ± 2.8	<0.05^c^
Mid-upper arm circumference (cm), mean ± SD	11.7 ± 2.3	14.2 ± 1.4	<0.05^c^
Triceps skin fold thickness (mm), median (range)	5.5 (4–10)	9 (5.3–13.1)	<0.05^a^
Subscapular skin fold thickness(mm), median (range)	4 (3.2–7.4)	8 (5.5–10.2)	<0.05^a^
**Biochemical markers levels**
Hemoglobin (g/dl), mean ± SD	10.2 ± 1.3	12.4 ± 1.0	<0.05^c^
Serum iron (μg/dl), median (range)	55.5 (35–84)	74.2 (46–105)	<0.05^a^
Serum ferritin (ng/dl), median (range)	43 (24–75)	76.5 (44–94)	<0.05^a^
Total serum protein (g/dl), mean ± SD	5.95 ± 1.0	7.0 ± 0.5	<0.05^c^
Serum albumin (g/dl), mean ± SD	3.6 ± 0.8	4.47 ± 0.7	<0.05^c^

*^a^Mann–Whitney U test*.

*^b^Chi-square test*.

*^c^t-test*.

All anthropometric measurements were significantly lower in the patients group than in the control group, as well as hemoglobin, serum iron, ferritin, and albumin levels.

Table 2 shows the distribution of cardiovascular defects in the patient group. Ventricular septal defect (VSD) was the most common cardiac lesion, whereas tetralogy of Fallot (TOF) was the most common cardiac lesion among cyanotic CHD.

**Table 2:** **Distribution of cardiovascular defects in children with CHD**.

Type of cardiac defects	No	%
**Acyanotic group (no. = 76)**
VSD	13	13.0
ASD	7	7.0
PDA	8	8.0
ASD + VSD	16	16.0
VSD + ASD + PDA	12	12.0
Coarcitation of aorta	4	4.0
Atrioventricular septal defect	10	10.0
Pulmonary stenosis	6	6.0
**Cyanotic group (no. = 24)**
Fallote (TOF)	12	12.0
Double outlet right ventricle (DORV)	5	5.0
Tricuspid atresia	3	3.0
Transposition of great vessels with atrial septostomy (Rashkind procedure)	4	4.0
Total	100	100

Table 3 shows the nutritional status for the case and control group. The overall prevalence of malnutrition reached 84.0% in cases compared to 20% for the control group. In children with CHD, prevalence of wasting (low WHZ) and stunting (low HAZ) were significantly higher compared with the control group. Also, the relative proportion of children with severe malnutrition was significantly higher in patients with CHD.

**Table 3:** **Nutritional status in patients and controls**.

	Cases (no. = 100)	Controls (no. = 100)	*p*-Value
**Nutritional status, *n*(%)**
Normal	16 (16%)	80 (80%)	<0.05
Malnutrition	84 (84%)	20 (20%)	
**Patterns of malnutrition, *n*(%)**
Under weight (WAZ ≤−2)	12 (14.3%)	4 (20%)	<0.05
Wasting (WHZ ≤−2)	20 (23.8%)	2 (10%)	<0.05
Stunting (HAZ ≤−2)	52 (61.9%)	14 (70%)	<0.05
**Degree of malnutrition, *n*(%)**
Moderate	24 (28.57%)	19 (95%)	0.389
Severe	60 (71.43%)	1 (5%)	<0.001

In patients with CHD, stunting was proportionately higher (57.89%) in the acyanotic subgroup, while wasting was predominant (45.83%) in the cyanotic subpopulation. There was no difference between cyanotic and acyanotic patients regarding the severity of malnutrition (Table 4).

**Table 4:** **Nutritional status in patients with acyanotic and cyanotic CHD**.

	Acyanotic CHD (no. = 76)	Cyanotic CHD (no. = 24)	*p*-Value
**Nutritional status, *n*(%)**
Normal	12 (15.78%)	4 (16.66%)	>0.05
Malnutrition	64 (84.21%)	20 (83.33%)	
**Patterns of malnutrition, *n*(%)**
Under weight (WAZ ≤−2)	11 (14.47%)	1 (4.16%)	>0.05
Wasting (WHZ ≤−2)	9 (11.84%)	11 (45.83%)	<0.05
Stunting (HAZ ≤−2)	44 (57.89%)	8 (33.33%)	<0.05
**Degree of malnutrition, *n*(%)**
Moderate	20 (26.31%)	4 (16.66%)	>0.05
Severe	44 (57.89%)	16 (66.66%)	>0.05

Malnutrition correlated significantly with low hemoglobin level, low arterial oxygen saturation, heart failure, pulmonary hypertension, and poor dietary history (Table 5).

**Table 5:** **Predicators of malnutrition in children with CHD**.

Variable	OR (95: CI)	*p*-Value
Anemia	9.33 (3.08–23.2)	<0.05
Low arterial oxygen saturation	3.72 (1.82–7.88)	<0.05
Heart failure	4.71 (7.05–11.05)	<0.05
Poor dietary history	3.0 (1.43–6.34)	<0.05
Pulmonary hypertension	2.01 (1.13–3.59)	<0.05

## Discussion

Different types of cardiac malformation can affect nutrition and growth to varying degrees. The severity of malnutrition can range from mild under-nutrition to failure to thrive (4). Our study has been performed to evaluate growth and nutritional status of children with CHD.

The high prevalence of 84% in our CHD group demonstrates the importance of malnutrition in patients with CHD. Moreover, 71.4% of cases had severe malnutrition.

Previous reports showed that CHD-related malnutrition is particularly common in developing countries, but prevalence varies widely from 27% up to 90.4%. For instance, Turkey, Mehrizi, and Drash (11) reported a malnutrition prevalence of 27% in children with CHD, whereas a more recent Turkish study (12) described a prevalence of 85%. In South India, Vaidyanathan and his colleagues (13) reported a high prevalence of underweight (59.0%) in children with CHD. In Nigeria, Okoromah and his colleagues (5) conducted a case–control observational study to evaluate prevalence and predictors of malnutrition in children with uncorrected symptomatic CHD describing a prevalence of 90.4%.

As in the study of Okoromah et al. (5), the high prevalence of malnutrition in our study may be explained by several factors including the distribution pattern of cardiac lesions, the presence of severe complications of CHD, such as CHF, and the prolonged absence of surgical CHD correction. This study was conducted in the only tertiary teaching hospital in our governorate to which cases with severe CHD and its complications are likely to be referred to for evaluation and management. In the present study, left to right shunt lesions in association with predominantly moderate to severe CHF were the most common cardiac lesions. The prolonged duration of not surgically corrected CHD is more likely to be associated with chronic hypoxemia, chronic heart failure, and sub optimal dietary intake.

The relative proportions of underweight, stunting, and wasting in the patient group were 14.3, 61.9, and 23.8%, respectively. Okoromah and his colleagues (5) reported that the relative proportions of underweight, stunting, and wasting, were 20.5, 28.8, and 41.1%, respectively. Ratanachu and Pongdara study (14) found that relative proportions of underweight, stunting, and wasting in Thailand children with CHD were 28, 16, and 22%, respectively.

In agreement with the usual distribution of growth deficiency in the general pediatric population in Egypt (15, 16), stunting rather than wasting was found to be the most common type of malnutrition in our study. This is in contrast to the results reported by Okoromah et al. (5) and also the preponderance of underweight described in the study of Ratanachu and Pongdara (14).

In the present study, stunting was linked to acyanotic CHD, while wasting was associated with cyanotic CHD. Several studies of the patterns of malnutrition in CHD vary widely (4, 5, 17, 18). In contrast to our results, Okoromah and his colleagues (5) reported that children with acyanotic CHD were more likely to be wasted, while those with cyanotic CHD were more prone to stunting. Linde and colleagues (17) reported that both wasting and stunting were more common in cyanotic CHD than in acyanotic CHD. Also, Salzer and coworkers (18) observed a higher prevalence of wasting in acyanotic CHD associated with left to right shunts and heart failure compared with cyanotic CHD.

These differences in the pattern of malnutrition among cyanotic and acyanotic patients may be due to the significant left to right shunt lesions in the acyanotic group that are more likely to be associated with chronic CHF and recurrent chest infections. These two factors, together with delayed surgical correction, might lead to the stunting type of chronic malnutrition. Second, regional variations in the distribution of malnutrition may also contribute to these differences as stunting was the most common type of malnutrition in the general Egyptian pediatric population (15, 16). Finally, and this may be the most important aspect, previous studies differed from our study concerning their design, characteristics of the study populations, and reference growth standards used for classification of malnutrition.

In the present study, the predictors of malnutrition were anemia, low arterial oxygen saturation, heart failure, poor dietary history, and pulmonary hypertension.

In agreement with these findings, Okoromah et al. (5) found that the predictors of malnutrition included CHF, type of CHD, duration of symptoms, age under 5 years, anemia, low arterial oxygen saturation, and poor dietary fat intake.

In south India, Vaidyanthan et al. (13) reported CHF, older age at corrective intervention, and lower growth potential as predictors of malnutrition in children with CHD, whereas factors like cardiac diagnosis, dietary intake, and socioeconomic scale had no significant impact on nutritional status. However, in Mexico, Villasís-Keever et al. (19) found that risk factors associated with malnutrition were the presence of a cyanotic CHD, the lack of nutritional supplementation, and a greater number of family members.

## Conclusion

Malnutrition is a very common complicating problem in children with symptomatic CHD, predicted by the presence of anemia, low arterial oxygen saturation, heart failure, poor dietary history, and pulmonary hypertension.

Based on the results of our study, an aggressive nutritional optimization for children with CHD should be considered, while efforts for early corrective interventions should be intensified. Future research should explore the short- and long-term impacts of improved nutrition on morbidity and mortality before and after corrective interventions for CHD.

## Author Contributions

All authors contributed to the current work as follows: BH, the first author, SM, and EA suggested the idea of the research and designed the work plan. The Egyptian authors contributed to the collecting of patients and interpreting and analyzing their data. BH and SM wrote and revised the paper, the last author, Professor OG, interpreted the results and made the final revision of this article before submission.

## Conflict of Interest Statement

The authors declare that there is no potential conflicts of interest exist with respect to the research, authorship, and/or publication of this article.
